# Pre-hospital delay and its influencing factors in patients with acute ischemic stroke: a cross-sectional study based on the health ecology model

**DOI:** 10.3389/fneur.2026.1789311

**Published:** 2026-05-13

**Authors:** Juan Wei, Qiaowei Li, Xing Liu, Lingling Ji, Cuiyun Zhang

**Affiliations:** 1 Neuromedical Center Ward II (Stroke Center), Geriatric Medicine Institute, The Affiliated Panyu Central Hospital of Guangzhou Medical University, Guangzhou, China; 2 Neuromedical Center Ward II (Stroke Center), The Affiliated Panyu Central Hospital of Guangzhou Medical University, Guangzhou, China

**Keywords:** acute ischemic stroke, cerebral infarction, influencing factors, pre-hospital, transient ischemic attack

## Abstract

**Background and objective:**

The treatment of acute ischemic stroke (AIS) is time-dependent, and pre-hospital delays remain a significant barrier to effective stroke management worldwide. Previous studies have often focused on isolated factors; however, the problem is multifaceted. This study lies in applying the Health Ecology Model as a comprehensive framework to systematically investigate the multifaceted factors (Intrapersonal, Interpersonal, Community, and Policy environment levels) associated with pre-hospital delay among AIS patients.

**Methods:**

A cross-sectional study was conducted, we consecutively enrolled 439 AIS patients admitted to the stroke center of a tertiary hospital in Guangzhou between January and December 2024. Data were collected through structured questionnaires and medical records, specifically aligned with the constructs of the Health Ecology Model. Measures included Intrapersonal level: Individual characteristics (e.g., number of stroke occurrences, symptoms at onset, mode of onset) and Behavioral and psychological factors (e.g., number of physical examination, Health literacy). Interpersonal level: family function (Family APGAR Index) and social support (Social Support Rating Scale). Community level: Living and working conditions (e.g., Employment status, Present residence). Policy environment level (e.g., Payment method, Awareness to call 120 for stroke emergency). Pre-hospital delay was defined as an onset-to-door time>6 h. Multivariable logistic regression identified independent influencing factors.

**Results:**

The pre-hospital delay rate was 54.44%. Significantly, factors from multiple levels of the Health Ecology Model were independently associated with delay: Lower stroke awareness(OR = 5.414, 95% CI 2.291–12.794), Perception of symptom severity (OR = 31.798, 95% CI 13.119–77.077), limb weakness /numbness (OR = 3.661, 95% CI 1.221–10.979), lower health literacy (OR = 1.064, 95% CI 1.041–1.088), poorer family function (OR = 1.545, 95% CI 1.138–2.097), lower social support (OR = 1.466, 95% CI 1.322–1.627), and stroke onset during the night (OR = 0.160, 95% CI 0.064–0.401),which increased the odds of delay.

**Conclusion:**

Pre-hospital delay is highly prevalent and is influenced by a complex interplay of factors across individual, family, and systemic levels, as elucidated by the Health Ecology Model. Our findings highlight the critical need to move beyond patient education alone and implement integrated, multi-level interventions. Public health campaigns should target both patients and their families to improve symptom recognition and health literacy. Concurrently, healthcare systems must be optimized, for instance by addressing barriers to after-hours care and strengthening pre-hospital pathways to ensure rapid triage to comprehensive stroke centers, ultimately improving access to timely revascularization therapies.

## 1 Introduction

Acute Ischemic Stroke (AIS) remains a leading global cause of death and disability, imposing a substantial burden on healthcare systems worldwide, particularly in aging populations like China’s (1–4). While the efficacy of acute reperfusion therapies—intravenous thrombolysis (IVT) and mechanical thrombectomy (MT)—is well-established, their success is critically time-dependent (5–7).

IVT with recombinant tissue-type plasminogen activator (rt-PA) is typically administered within 4.5 h of symptom onset. For eligible patients with large vessel occlusions, MT can be performed up to 24 h based on advanced neuroimaging selection (e.g., CT Perfusion) (8, 9). Despite the established effectiveness of these treatments, their utilization remains disappointingly low in China, with an IVT rate of only approximately 5.64% (9). A critical barrier is the profound pre-hospital delay, evidenced by a median onset-to-door time (OTD) of 23.83 h, which far exceeds these narrow windows (10). Extensive prior research has identified isolated factors contributing to pre-hospital delay, including lack of stroke symptom recognition, failure to perceive symptom severity, and inadequate emergency service utilization (11–14). However, these studies have not comprehensively examined organizational factors. A recent review by Botelho et al. (15) specifically addresses this aspect, highlighting the role of organizational barriers in pre-hospital delay and underscoring the need for further investigation into systemic influences. Traditional analyses focusing on single-level factors (e.g., solely patient-level or provider-level) have proven insufficient to fully explain the multifactorial nature of help-seeking behavior and delays. This gap necessitates a more holistic and integrative analytical framework.

This study is motivated by the critical need to move beyond a siloed examination of pre-hospital delay factors. To address this complexity, we employ the Health Ecology Model (16). This model provides a comprehensive, multi-level framework to understand health behaviors by examining the synergistic interplay between.

Intrapersonal factors (e.g., Number of stroke occurrence, Symptoms at onset, health literacy).

Interpersonal factors (e.g., Family function, Social support).

Institutional/Community factors (e.g., income, employment, residence, health insurance type, and awareness of emergency systems), and awareness of emergency systems (e.g., calling 120 [the national emergency medical services hotline in China]).

Policy factors (e.g., stroke networks).

The rationale for using this model is robust: it is uniquely suited to capture the layered determinants of help-seeking behavior that collectively contribute to delay. By applying this model, we aim to generate a systems-level understanding of the problem, which is a prerequisite for designing coordinated, multi-faceted, and effective interventions that simultaneously target barriers across different ecological levels.

Therefore, this study aimed to: (1) investigate the rate of pre-hospital delay among patients with acute ischemic stroke, and (2) examine the influencing factors based on the Health Ecology Model framework, with the goal of identifying targets for future interventions to reduce delay and improve stroke outcomes.

## 2 Methods

### 2.1 Study design and setting

A cross-sectional study was conducted at the stroke center of a tertiary hospital in Guangzhou, China. This facility serves as a regional referral center for acute cerebrovascular diseases. Participant recruitment and data collection took place between January 2024 and December 2024.

### 2.2 Study objectives and hypotheses

#### 2.2.1 Primary objective

To comprehensively analyze the factors influencing pre-hospital delay among AIS patients by applying the multi-level framework of the Health Ecology Model.

#### 2.2.2 Secondary objectives

To quantify the rate of pre-hospital delay (OTD > 6 h) in the studied cohort.

To identify specific factors associated with delay within each level of the model (intrapersonal, interpersonal, community, policy).

To determine the relative contribution of factors from different model levels to the risk of delay.

#### 2.2.3 Prespecified hypothesis

We hypothesize that pre-hospital delay is not determined by isolated factors but is the result of a complex interaction of determinants across all levels of the Health Ecology Model. Specifically, we hypothesize that after adjusting for clinical variables, factors such as low stroke awareness (intrapersonal), poor social support (interpersonal), and rural residence (community) will be independently associated with a higher likelihood of delay.

### 2.3 Participants

#### 2.3.1 Inclusion criteria

Eligible patients met all of the following criteria: (1) primary diagnosis of Acute Ischemic Stroke (AIS), confirmed by head CT or MRI according to the Chinese Guidelines for the Diagnosis and Treatment of Acute Ischemic Stroke 2023 (9); (2) age ≥18 years; (3) sufficient cognitive and communicative ability, indicated by a Token Test score >17, to ensure reliable comprehension of questionnaire items; (4) ability to provide informed consent by themselves or through a legally authorized representative.

#### 2.3.2 Exclusion criteria

Patients were excluded based on the following criteria: (1) presence of severe comorbidities (e.g., end-stage renal disease, terminal cancer) or a pre-existing major mental disorder (e.g., severe dementia, schizophrenia) that could confound the assessment of delay reasons or impair interview reliability; (2) severe hearing or visual impairment precluding independent completion of assessments; (3) unstable clinical condition (e.g., significantly impaired consciousness (GCS < 12), requiring intensive care) or severe physical weakness that would prevent cooperation with a 15-min interview; (4) inability to determine the approximate time of symptom onset.

### 2.4 Theoretical framework: variable definition and measurement

The Health Ecology Model was adopted as the comprehensive framework to guide the analysis of pre-hospital delay, a behavior influenced by multi-level determinants. This model examines the interplay across five levels: (1) intrapersonal, (2) interpersonal, (3) institutional/organizational, (4) community, and (5) policy. Our study operationalized variables from five levels.

#### 2.4.1 Primary outcome

The primary outcome was pre-hospital delay. Pre-hospital delay was defined as the time from symptom onset to hospital arrival (onset-to-door time, OTD). It is important to note that this study focuses on patient- and caregiver-level factors influencing care-seeking behavior; we did not evaluate emergency medical services (EMS) system performance. According to the Chinese Guidelines for the Diagnosis and Treatment of Acute Ischemic Stroke 2023 (17), patients with onset within 6 h can be considered for intravenous thrombolysis with urokinase based on the criteria for indications and contraindications. A pre-hospital delay of more than 6 h was defined as significant because it exceeds the standard time window for effective reperfusion therapies, particularly mechanical thrombectomy, which is most beneficial within 6 h of symptom onset for anterior circulation large vessel occlusion. Thus, 6 h was chosen as the cutoff point, and also as a binary variable (OTD > 6.0 h, Yes/No).

#### 2.4.2 Exposures, predictors, and potential confounders

All predictors and potential confounders were selected *a priori* based on the Health Ecology Model and are detailed in Table 1.

**Table 1:** Study variables mapped to the health ecology model framework.

Level	Variables	Measurement/instrument
Intrapersonal	Demographics and clinical traits	Age, gender, education, stroke history, symptoms at onset, mode of onset, time of onset, location of onset,changes in symptoms before hospitalization. (General Questionnaire)
	Psychological and behavioral factors	physical examination history, health literacy (Stroke Health Literacy Scale), awareness of stroke symptoms, severity perception (General Questionnaire)
Interpersonal	Social network and function	Marital status, living situation, family function (Family APGAR Index), social support (SSRS)
Institutional/Community	Living and economic conditions	Monthly income, residence location (urban/rural), employment status (General Questionnaire)
	Health system awareness	awareness to call emergency number 120, awareness of stroke map (General Questionnaire)
Policy-level factors	Health insurance type	Payment method(General Questionnaire)
Outcome	Pre-hospital delay	Onset-to-door time (minutes), and OTD > 6.0 h (dichotomous)

### 2.5 Study instruments and measures

For each variable of interest, data sources and detailed assessment methods are described below. All data were collected by trained researchers using standardized procedures to ensure comparability across all participants. As this was a single-group cross-sectional study, the same assessment methods were applied uniformly to all enrolled patients.

#### 2.5.1 General information questionnaire

A structured, self-designed questionnaire was used to collect data on socio-demographic and clinical variables. The sources and measurement methods for each variable are as follows:

Socio-demographic data: Including gender, age, education level, marital status, insurance type, monthly household income per capita, employment status, current residence (urban/rural), living situation, and physical examination history. These data were obtained via patient or proxy self-report.

Clinical characteristics: Including stroke history, location at onset, time of onset, mode of onset (sudden/gradual), initial symptom perception and identification, perception of symptom severity, and changes in symptoms before hospitalization. These were assessed through medical record review and patient/family interview.

Stroke awareness items: Two specific items were included: (1) “Awareness to call the emergency number 120 for stroke” (yes/no), reflecting knowledge of China’s universal emergency number; and (2) “Awareness of the stroke map” (yes/no), indicating whether the participant knew about the regional network of certified stroke centers.

#### 2.5.2 Stroke patient health literacy scale

Health literacy was measured using the validated Stroke Patient Health Literacy Scale developed by Liu et al. (18). This instrument includes 20 items across three dimensions: basic knowledge and concepts, healthy lifestyle and behaviors, and basic skills. Each item is rated on a 5-point Likert scale from 1 (“not important”) to 5 (“very important”). Total scores range from 20 to 100, with higher scores indicating better health literacy. In the original validation study, the Cronbach’s *α* was 0.932, and test–retest reliability ranged from 0.889 to 0.936 across subscales. In this study, the overall Cronbach’s α was 0.936.

#### 2.5.3 Family APGAR index

Perceived family function was assessed using the Family APGAR Index (19), a 5-item instrument scored from 0 to 2 per item. Total scores range from 0 to 10, with higher scores reflecting better family functioning. Scores of 7–10 indicate highly functional families, 4–6 indicate moderate family dysfunction, and 0–3 indicate severe family dysfunction. In this study, Cronbach’s *α* was 0.75.

#### 2.5.4 Social support rating scale (SSRS)

Social support was evaluated with the SSRS (20), which contains 10 items covering three dimensions: objective support (3 items), subjective support (4 items), and support utilization (3 items). Scoring rules are clearly defined per item, including single-choice questions (scored 1–4) and multiple selections (scored by number of sources). The original scale reported a Cronbach’s *α* of 0.99. In this study, internal consistency was 0.812.

### 2.6 Data collection and quality control

Trained researchers administered all instruments using a standardized protocol to minimize bias. Eligible patients were identified upon admission to the stroke center and approached within 7 days of hospital admission to ensure accurate recall of pre-hospital events while prioritizing clinical stability. After obtaining informed consent, Scales included in this study were administered via face-to-face interviews conducted within 24–72 h of admission, once the patient’s condition had stabilized and they were able to participate meaningfully. For patients with aphasia or severe neurological deficits, family caregivers who were primarily responsible for the patient’s care were interviewed as proxies, with this documented in the dataset. To ensure assessment comparability and reduce information bias, researchers read questions verbatim without interpretation to participants who required assistance. To enhance accuracy of recall for symptom onset severity, patients (or families if the patient was unable) were asked: “Did you think your symptoms were severe when they first appeared?” This was intended to capture the initial perception of threat rather than a retrospective judgment influenced by subsequent diagnosis.

The time of symptom onset was rigorously defined as the last time the patient was known to be at their usual neurological status. For patients awakening with symptoms, onset time was recorded as when they were last known to be well (e.g., when they went to sleep). This was confirmed through simultaneous interviews with patients and witnesses whenever possible.

All questionnaires were reviewed for completeness immediately after collection. Among 445 questionnaires distributed, 439 were valid and included in the analysis, yielding a response rate of 98.65%.

### 2.7 Sample size calculation

The study sample size was determined based on the principle of including at least 10 participants per variable (EPV) for multivariate logistic regression analysis. With approximately 20 primary variables derived from the ecological model, a minimum sample size of 200 was required. Accounting for potential missing data and invalid responses, we aimed to recruit 440 participants, which also aligns with sample sizes reported in similar methodological studies focusing on pre-hospital delay.

## 3 Handling of quantitative variables

In the analysis, continuous variables were described using means ± standard deviations or medians with interquartile ranges, depending on their distribution. For the primary outcome—pre-hospital delay—onset-to-door time (OTD) was analyzed as dichotomized into delay (OTD > 6 h) vs. no delay (OTD ≤ 6 h) based on the therapeutic window for thrombolysis. Other quantitative variables, such as health literacy and social support scores, were treated as continuous in primary analyses. However, for clinical interpretation, some variables (e.g., Family APGAR) were also categorized into predefined groups as recommended by their original validation studies. All grouping choices were made *a priori* based on clinical relevance or conventional cut-offs described in the literature.

### 3.1 Statistical analysis

Data entry was verified independently by two researchers to ensure accuracy. All statistical analyses were performed using SPSS software (version 23.0). Descriptive statistics were presented as mean ± standard deviation for normally distributed continuous variables, median (interquartile range) for non-normally distributed data, and as frequency (percentage) for categorical variables. Normality was assessed using the Shapiro–Wilk test.

#### 3.1.1 Methods to control for confounding

Univariate analyses were first conducted to identify variables associated with pre-hospital delay. Independent-samples t-tests or Mann–Whitney *U* tests were used for continuous variables, and Chi-square (χ^2^) or Fisher’s exact tests were used for categorical variables. Variables with *p* < 0.05 in univariate analyses were included as candidates in multivariate logistic regression to control for potential confounding. The final model was constructed using stepwise backward elimination (removal threshold *p* > 0.05), with pre-hospital delay (OTD > 6 h) as the dependent variable. Results are presented as adjusted odds ratios (aOR) with 95% confidence intervals (CI). Model fit was evaluated using the Hosmer-Lemeshow test.

#### 3.1.2 Subgroup and interaction analyses

Subgroup analyses were performed based on clinically relevant factors such as gender, age group (<60 vs. ≥60 years. Effect modification was assessed by including interaction terms (e.g., age × health literacy, family function × social support) in the logistic regression model. A significance level of *p* < 0.10 was used to identify potential interactions.

#### 3.1.3 Handling of missing data

Missing data were handled through multiple imputation using chained equations (MICE) for variables with <10% missingness. Complete-case analysis was performed for validation. Variables with extensive missing values (>20%) were excluded from multivariate models.

#### 3.1.4 Consideration of sampling strategy

Given the use of a convenience sampling strategy at a single center, the analysis did not incorporate sampling weights. However, we performed multilevel logistic regression to account for potential clustering effects related to admission period or seasonal variations.

#### 3.1.5 Sensitivity analyses

Sensitivity analyses were conducted to assess the robustness of the results. These included.

Using different thresholds for pre-hospital delay (e.g., OTD > 6 h).

Repeating the multivariate analysis with enter method instead of stepwise selection.

Excluding patients with unclear symptom onset time or those who awoke with symptoms.

A two-tailed *p*-value <0.05 was considered statistically significant, except for interaction terms where *p* < 0.10 was used.

## 4 Results

### 4.1 Participant flow and recruitment

**Figure 1:**
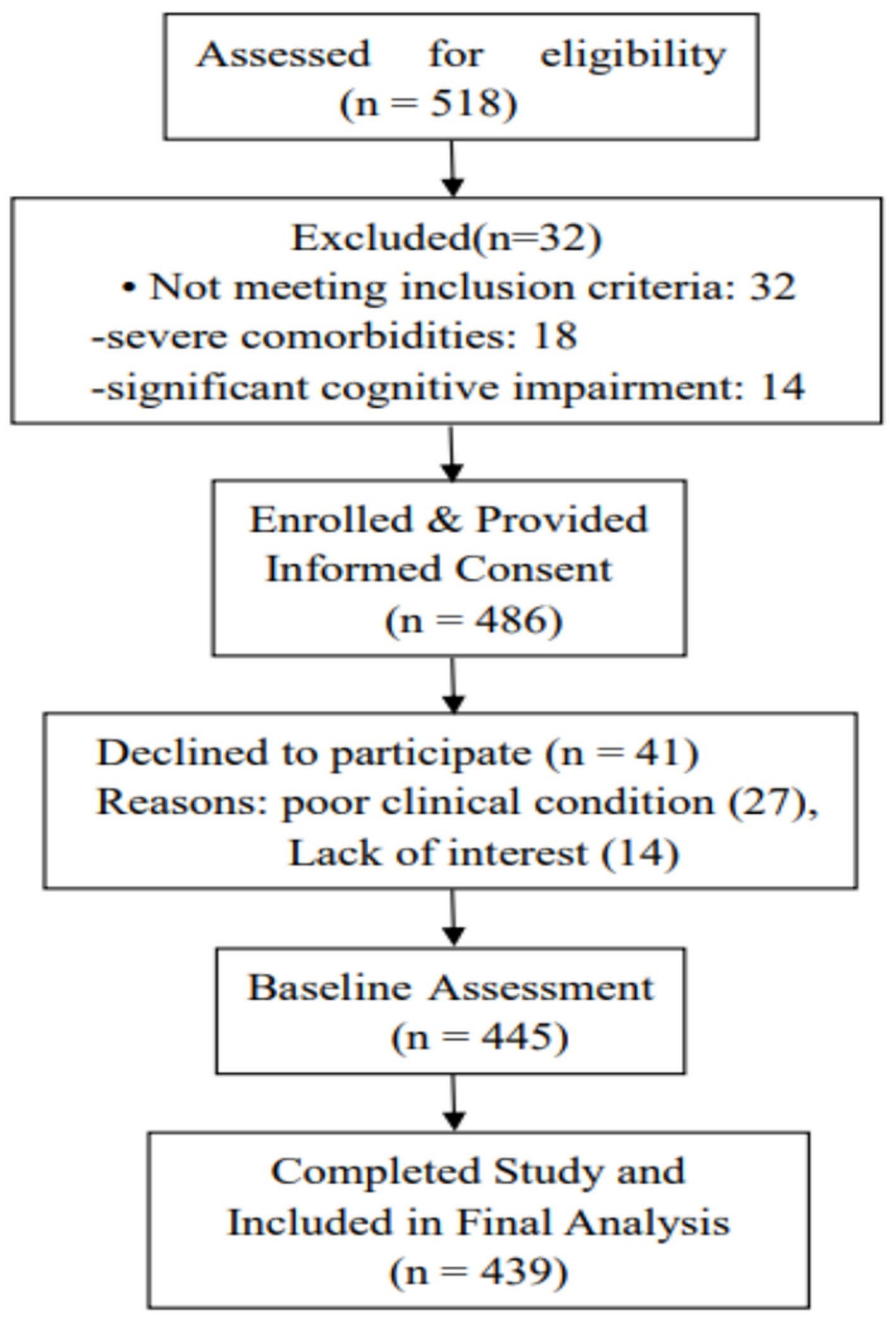
Flow diagram of participant recruitment and inclusion.

A total of 518 consecutive AIS patients admitted to the stroke center between January and December 2024 were initially screened for eligibility using a convenience sampling method. Of these, 73 patients were excluded: 32 did not meet inclusion criteria (18 due to severe comorbidities, 14 with significant cognitive impairment), and 41 declined to participate (27 due to poor clinical condition, 14 refused consent). Consequently, 445 patients were enrolled and completed the baseline assessment within 3 days of hospital admission to ensure accurate recall of pre-hospital events while prioritizing clinical stability. After excluding 6 patients with incomplete data, 439 patients were included in the final analysis, yielding a response rate of 98.65%. A detailed flow diagram of participant selection is presented in Figure 1

### 4.2 Analysis of pre-hospital delay in AIS patients

In this study, the median onset-to-door time (OTD) for AIS patients was 18 h. Based on the OTD time, the 439 patients were divided into two groups: the ≤6 h group (non-pre-hospital delay group) and the >6 h group (pre-hospital delay group). There were 200 patients in the ≤6 h group and 239 in the >6 h group, with a pre-hospital delay rate of 54.44% among AIS patients.

### 4.3 Univariate analysis of pre-hospital delay in AIS patients

A univariate analysis of pre-hospital delay in AIS patients based on different characteristics is shown in Table 2.

**Table 2:** Univariate analysis of pre-hospital delay in AIS patients.

Variables	Categories	Non-pre-hospital delay group (*n* = 200)	Pre-hospital delay group (*n* = 239)	Statistical value	*p*
Gender, *n* (%)	Male	134 (67.0)	169 (70.7)	*x*^2^ = 0.701	0.409
	Female	66 (33.0)	70 (29.3)		
Age (years), *n* (%)	31–60	141 (70.5)	150 (62.8)	*x*^2^ = 3.465	0.063
	≥60	59 (29.5)	89 (37.2)		
Educational status, *n* (%)	Middle school and below	96 (48.0)	110 (46.0)	*x*^2^ = 0.510	0.610
	high school or vocational high school	83 (41.5)	102 (42.7)		
	College degree and above	21 (10.5)	27 (11.3)		
Marital status, *n* (%)	Married	166 (83.0)	189 (79.1)	*x*^2^ = 1.082	0.298
	Widowed/divorced	34 (17.0)	50 (20.9)		
Payment method, *n* (%)	Non-self-paid	157 (78.5)	185 (77.4)	*x*^2^ = 0.076	0.783
	Self-paid	43 (21.5)	54 (22.6)		
Family monthly income per capita, *n* (%)	Less than 3,000	68 (34.0)	73 (30.5)	*x*^2^ = 0.675	0.500
	3,000–4,999	93 (46.5)	117 (49.0)		
	5,000 or higher	39 (19.5)	49 (20.5)		
Employment status, *n* (%)	Employed	39 (19.5)	48 (20.1)	*x*^2^ = 0.023	0.879
	Unemployed	161 (80.5)	191 (79.9)		
Present residence, *n* (%)	Urban areas	110 (55.0)	114 (47.7)	*x*^2^ = 6.838	0.033
	Rural areas	66 (33.0)	74 (31.0)		
	Urban–rural integration area	24 (12.0)	51 (21.3)		
Living situation, *n* (%)	Living alone	25 (12.5)	32 (13.4)	*x*^2^ = 0.076	0.783
	Living with others	175 (87.5)	207 (86.6)		
Number of physical examination, *n* (%)	Once per year or more	113 (56.5)	107 (44.8)	*x*^2^ = 8.567	0.003
	Once every few years	45 (22.5)	51 (21.3)		
	Never	42 (21.0)	81 (33.9)		
Stroke history, *n* (%)	Once	134 (67.0)	181 (75.7)	*x*^2^ = 4.096	0.043
	Two times or more	66 (33.0)	58 (24.3)		
Location of onset, *n* (%)	Home	165 (82.5)	196 (82.0)	*x*^2^ = 0.018	0.893
	Other places	35 (17.5)	43 (18.0)		
Time of onset, *n* (%)	06:01 ~ 18:00	114 (57.0)	89 (37.2)	*x*^2^ = 17.105	<0.001
	18:01 ~ 6:00	86 (43.0)	150 (62.8)		
Mode of onset, *n* (%)	Sudden	168 (84.0)	162 (67.8)	*x*^2^ = 15.343	<0.001
	Gradual	32 (16.0)	77 (32.2)		
Symptoms at onset, *n* (%)	Dizziness or unsteady gait			*x*^2^ = 6.216	0.013
	Yes	48 (24.0)	35 (14.6)		
	No	152 (76.0)	204 (85.4)		
	Limb weakness or numbness, falls			*x*^2^ = 16.453	<0.001
	Yes	178 (89.0)	176 (73.6)		
	No	22 (11.0)	63 (26.4)		
	Speech dysfunction			*x*^2^ = 1.977	0.160
	Yes	82 (41.0)	114 (47.7)		
	No	118 (59.0)	125 (52.3)		
	Facial numbness or mouth corner deviation			*x*^2^ = 1.024	0.312
	Yes	25 (12.5)	38 (15.9)		
	No	175 (87.5)	201 (84.1)		
	Vision blurred or loss			*x*^2^ = 0.045	0.831
	Yes	14 (7.0)	18 (7.5)		
	No	186 (93.0)	221 (92.5)		
**S**ymptom changes before hospitalization	Yes	46 (23.0)	94 (39.3)	*x*^2^ = 13.369	<0.001
	No	154 (77.0)	145 (60.7)		
Perception of symptom severity	Believe it is very serious and need to go to the hospital immediately	163 (81.5)	41 (17.2)	*x*^2^ = 181.229	<0.001
	Believe it is not serious and did not take it seriously	37 (18.5)	198 (82.8)		
Awareness of stroke symptoms	Yes	106 (53.0)	50 (20.9)	*x*^2^ = 48.914	<0.001
	No	94 (47.0)	189 (79.1)		
Awareness to call 120 for stroke emergency	Yes	41 (20.5)	39 (16.3)	*x*^2^ = 1.278	0.258
	No	159 (79.5)	200 (83.7)		
Awareness of stroke map	Yes	18 (9.0)	24 (10.1)	*x*^2^ = 0.137	0.712
	No	182 (91.0)	215 (89.9)		
Health literacy, (^−^*x* ± s)		65.21 ± 18.32	51.50 ± 14.91	*t* = 6.451	<0.001
Family function, (^−^*x* ± s)		5.19 ± 1.54	4.21 ± 1.21	*t* = 7.478	<0.001
Social support, (^−^*x* ± s)		42.88 ± 7.03	35.85 ± 7.47	*t* = 19.196	<0.001

(1) The ‘Not married’ category includes participants who were single (never married), widowed, or divorced, due to the small number of single participants in this predominantly older adult population; (2) The ‘Not employed’ category includes retired participants, homemakers/stay-at-home parents, and those who were unemployed and seeking work; (3) Present residence categorized as urban, rural, or urban–rural integration area (a transitional zone analogous to suburbs, but with distinct mixed administrative and socio-economic features which was classified according to China’s national standard ‘Provisions on the Statistical Delineation of Urban and Rural Areas’ (Guo Han [2008] No. 60)).

### 4.4 Multivariate analysis of pre-hospital delay in AIS patients

Variable assignment is shown in Table 3. Using pre-hospital delay as the dependent variable and factors with statistical significance in the univariate analysis as independent variables, the analysis found that Awareness of stroke symptoms, Perception of symptom severity, Limb weakness or numbness, Health literacy, Family function, Social support and Onset time were independent influencing factors. Detailed information can be found in Table 4.

**Table 3:** Variable assignment table.

Variable	Assignment
Present residence	Urban areas = (0, 0), Rural areas = (0, 1), Urban–rural integration area = (1, 0)
Number of physical examination	Never = 1, Once every few years = 2, Once per year or more = 3
Number of stroke occurrence	Once = 0; Two times or more = 1
Onset time	06:00 ~ 18:00 = 0; 18:01 ~ 6:00 = 1
Mode of onset	Sudden = 0; Gradual = 1
Dizziness or unsteady gait	No = 0; Yes = 1
Limb weakness or numbness, falls	No = 0; Yes = 1
**S**ymptom changes from onset to hospitalization	No = 0; Yes = 1
Perception of symptom severity	It is not serious = 0; It is very serious = 1
Awareness of stroke symptoms	No = 0; Yes = 1
Health literacy	Original value
Family function	Original value
Social support	Original value

**Table 4:** Multivariate analysis of pre-hospital delay in AIS patients.

Variable	*B*	SE	Wald	*p*	OR	95% CI
Constant	−23.342	2.599	80.650	<0.001	0.000	
Awareness of stroke symptoms	1.689	0.439	14.820	<0.001	5.414	[2.291, 12.794]
Perception of symptom severity	3.459	0.452	58.645	<0.001	31.798	[13.119, 77.077]
Limb weakness or numbness, falls	1.298	0.560	5.362	0.021	3.661	[1.221, 10.979]
Onset time	−1.834	0.469	15.281	<0.001	0.160	[0.064, 0.401]
Health literacy	0.062	0.011	30.523	<0.001	1.064	[1.041, 1.088]
Family function	0.435	0.156	7.788	0.005	1.545	[1.138, 2.097]
Social support	0.383	0.053	52.298	<0.001	1.466	[1.322, 1.627]

*R*^2^ = 0.684, ad *R*^2^ = 0.678.

## 5 Discussion

### 5.1 High rate of pre-hospital delay in AIS patients

This study revealed a pre-hospital delay rate of 54.44% among AIS patients, with a median onset-to-door time of 18 h. This finding is consistent with the widespread nature of this challenge, as reported in previous literature (8, 21, 22). For instance, Hoa et al. (23) reported similar delays, and a large-scale survey found that nearly 50% of patients sought medical care more than 24 h after symptom onset (10). The high delay rate observed in this study that pre-hospital delay remains a significant barrier to achieving timely reperfusion therapy, despite improvements in reducing in-hospital treatment times (e.g., door-to-needle time). Therefore, identifying and addressing the factors influencing patient delays is a critical and urgent issue to improve clinical outcomes.

### 5.2 The influencing factors of pre-hospital delay in AIS patients

#### 5.2.1 Awareness of stroke symptoms

Our study identified lower awareness of stroke symptoms as a significant factor associated with pre-hospital delay. This finding aligns with previous reports emphasizing that deficient symptom knowledge often leads to hesitation in seeking emergency care (24, 25). The effectiveness of public education campaigns, such as those employing the FAST mnemonic, in improving recognition and prompting timely action has been demonstrated elsewhere (26, 27). Our results reinforce the critical need to integrate such targeted educational strategies, particularly for high-risk populations and their families, to create a more responsive care-seeking environment. Therefore, enhancing stroke literacy should be considered a fundamental component of efforts to optimize pre-hospital pathways and improve acute stroke outcomes.

#### 5.2.2 Perception of symptom severity

A central finding of our study is the profound impact of symptom severity perception on pre-hospital delay, which demonstrated a very strong positive association in our multivariate analysis. This result aligns with previous reports (28). Critically, our analysis reveals that an individual’s perception of severity is a powerful and independent predictor of delay, even after accounting for other factors like social support and health literacy. This suggests that a patient’s intrinsic assessment of their symptoms can override external influences and systemic barriers in the decision-making process. Therefore, while improving resource allocation and public awareness remains important, our findings highlight an urgent need to develop novel, perception-focused interventions. Clinical strategies should prioritize empowering at-risk individuals and their families to correctly interpret and act upon early warning signs, moving beyond general education to target the psychological and cognitive mechanisms that shape severity perception. This refocusing on the patient’s immediate appraisal of threat offers a new pathway for nursing intervention to reduce care-seeking delays.

#### 5.2.3 Limb weakness or numbness, falls

Notably, our study identified that the absence of limb weakness or numbness was independently associated with a higher likelihood of pre-hospital delay. While these classic stroke symptoms are highly recognizable and often trigger urgent care-seeking, our findings reveal a critical nuance: their absence does not eliminate the risk of delay but rather conceals it within a more complex cognitive and behavioral profile. This finding may be particularly relevant in the context of posterior circulation strokes, which often present with less recognizable symptoms such as vertigo, dizziness, gait disturbance, or visual deficits rather than typical limb weakness. Previous studies have consistently demonstrated that posterior circulation stroke is associated with longer pre-hospital delays due to atypical presentations and lower public awareness of these symptoms (29, 30). Patients and even initial healthcare contacts may misinterpret these non-motor symptoms as benign conditions (e.g., vestibular disorders), leading to delayed care-seeking behavior. Our results underscore the need for public education campaigns that emphasize the diverse symptom presentations of stroke, including those affecting the posterior circulation.

This result provides a crucial refinement to the health belief model in the stroke context. It indicates that public education campaigns like “F. A. S. T.,” though valuable (31–33), might inadvertently create a dependency on these specific deficits. Patients experiencing non-motor or “atypical” symptoms may fail to recognize their condition as an emergency, leading to dangerous normalization of their symptoms. Therefore, our research underscores an urgent need to expand public symptom awareness beyond the F. A. S. T. framework to include a broader range of warning signs.

Furthermore, our ecological approach suggests that future interventions must be stratified not only by population risk but also by symptom presentation. For vulnerable groups, such as those in remote areas with limited health access, education must explicitly address the diversity of stroke symptoms, empowering individuals to act even in the absence of limb weakness. This shift from a symptom-specific to a symptom-inclusive alert strategy represents a necessary evolution in nursing education and public health communication.

#### 5.2.4 Health literacy

In our study, lower health literacy emerged as a significant and independent factor associated with pre-hospital delay among AIS patients. This finding reinforces its pivotal role in the early recognition of stroke symptoms and the decision-making process to seek urgent medical care (34–37).

Unlike studies that treat health literacy merely as an individual trait, our analysis within the Health Ecological Model demonstrates that its impact persists even after accounting for social and environmental factors. This suggests that improving a patient’s ability to access, understand, and apply health information is a necessary and direct pathway to reducing delays.

Our results argue for the integration of structured health literacy assessments into routine clinical practice, especially for high-risk populations. Interventions should move beyond general public education to include targeted, low-literacy communication strategies that help patients and families recognize stroke symptoms and understand the urgency of immediate action. Enhancing health literacy at both individual and community levels represents a practical and evidence-based strategy to improve stroke outcomes.

#### 5.2.5 Family function

Our study provides robust evidence that poorer family function is a significant and independent predictor of pre-hospital delay in AIS patients. While the general importance of family support is recognized (14, 38, 39), our analysis within the Health Ecological Model offers a critical advancement: the influence of family function remains significant even after controlling for individual-level factors such as health literacy and symptom awareness.

This finding underscores that a well-functioning family unit—characterized by effective communication, clear emergency roles, and collaborative decision-making—acts as a vital facilitator in the acute phase of stroke, often compensating for individual limitations. In clinical terms, this means that a patient’s likelihood of receiving timely care depends not only on their own knowledge but also on the capability and preparedness of their family network.

Therefore, our research argues strongly for integrating family-centric assessments and interventions into standard stroke care protocols. This could involve screening for family dysfunction in high-risk patients and providing targeted education and role-based emergency planning for families. By recognizing and strengthening the family as a foundational unit of acute care response, clinicians can address a key ecological barrier to treatment delay identified in our study.

#### 5.2.6 Social support

Our study identified lower social support as a significant and independent factor associated with pre-hospital delay in AIS patients. This finding highlights that beyond individual knowledge and family dynamics, the broader availability of emotional, informational, and practical support from one’s social network critically influences timely care-seeking behavior (11, 40).

Specifically, our results indicate that patients with stronger social networks were more likely to seek medical attention promptly following symptom onset. This suggests that emotional reassurance, informational guidance, and practical assistance from one’s social environment may help overcome barriers to care by reducing uncertainty and facilitating access to emergency services (41–43).

These findings emphasize the importance of assessing social support levels in stroke risk assessment protocols. For isolated or vulnerable patients, targeted interventions such as community health worker programs, caregiver education, and enhanced community-emergency service integration could help mitigate the impact of limited social support on treatment delays.

#### 5.2.7 The time of onset

Our study identified stroke onset during the night-time frame as a significant and independent risk factor for pre-hospital delay. This finding aligns with existing reports (28).

The strong association observed in our cohort may be attributed to the combined effect of reduced symptom awareness during sleep and limited immediate availability of assistance. Furthermore, patient and family reluctance to activate emergency services during nighttime hours likely contributes to treatment hesitancy.

These results highlight the need for targeted public health messaging that specifically addresses nocturnal stroke recognition and emphasizes the necessity of immediate emergency response regardless of the time of day. By recognizing temporal vulnerability as a modifiable risk factor, healthcare systems can develop more precise approaches to reducing stroke-related disability.

#### 5.2.8 Others

Although multiple sociodemographic and clinical factors demonstrated significant associations with pre-hospital delay in univariate analyses, most of these did not retain statistical significance in the multivariate model. This attenuation suggests that the effects of these variables may be mediated through more proximal determinants, particularly patients’ perception of symptom severity and their subsequent care-seeking behaviors. For instance, the apparent disadvantage observed in rural residents may primarily stem from lower health literacy or limited access to social support networks rather than geographic location itself.

These results underscore the complex interplay of factors influencing pre-hospital delay and highlight the value of using multivariate analytical approaches to identify the most direct and actionable intervention targets. The findings suggest that interventions focusing on improving health literacy, strengthening social support, and enhancing symptom recognition may benefit patients across different demographic and clinical profiles.

## 6 Limitations

Despite the valuable insights provided by this study, several limitations should be acknowledged: (1) The study population was drawn primarily from a single region or institution, which may limit the generalizability of the findings. (2) Some information relied on patient or caregiver recall, particularly regarding symptom perception and time to seek care, which may affect the accuracy of the data. (3) Elements such as healthcare accessibility and transportation barriers were not thoroughly quantified, potentially underestimating their impact on pre-hospital delay. (4) Fourth, our measurement of the “initial perception of symptom severity” is susceptible to recall bias. Although we specifically asked participants to recall their thoughts before the diagnosis was confirmed, their current knowledge of having had a stroke may have influenced their retrospective reporting. However, we attempted to mitigate this by interviewing patients as early as possible during their hospitalization and, when necessary, corroborating the account with family members who were present at onset. (5) We excluded patients with low GCS scores (<8) due to their inability to provide informed consent or complete the health literacy assessment. This exclusion may introduce selection bias and limit the generalizability of our findings. Patients with severe neurological impairment may represent a particularly vulnerable subgroup with distinct delay patterns—potentially experiencing longer pre-hospital delays due to inability to self-activate emergency services, greater reliance on bystander recognition and decision-making, and increased family deliberation time. Their exclusion likely means that our results underestimate the true extent of pre-hospital delay in the overall AIS population. Future studies specifically designed to capture delay patterns in severely affected patients, potentially using proxy respondents or alternative data sources, are needed to address this important knowledge gap. (6) Our employment status categorization did not distinguish between retired individuals, homemakers, and those who were truly unemployed, which may obscure important differences in socioeconomic circumstances and their potential influence on care-seeking behaviors. Future studies should employ more granular employment classifications.

## 7 Summary

This study demonstrates that the pre-hospital delay rate among patients with acute ischemic stroke (AIS) remains high and is influenced by multiple independent factors. Key determinants include the patient’s awareness of stroke symptoms, perception of symptom severity, limb weakness or numbness, level of health literacy, family function, social support, and time of symptom onset. Additionally, variables such as place of residence, frequency of physical examinations, number of previous stroke events, mode of onset, initial symptoms (e.g., dizziness or unsteady gait), and symptom progression from onset to hospital admission also contribute to delays in seeking medical care. Beyond individual patient factors, the efficiency of prehospital stroke care is critically dependent on well-organized treatment pathways. An effective prehospital stroke system typically encompasses several key components: public education for symptom recognition, emergency medical dispatch with stroke-specific protocols, prehospital stroke assessment tools (e.g., FAST, CPSS, or LAMS), prenotification of receiving hospitals, and direct transport to designated stroke centers capable of providing appropriate reperfusion therapies (44, 45). Integrated stroke systems of care have been shown to significantly reduce onset-to-treatment times and improve functional outcomes (46).

In the Chinese context, prehospital stroke care pathways are still evolving. Although urban emergency medical services (EMS) systems have established stroke protocols, utilization remains suboptimal—as evidenced by the low EMS activation rate in our cohort. Barriers to effective prehospital care in China include variable public awareness of stroke symptoms, inconsistent use of prehospital stroke scales by first responders, and regional disparities in access to stroke centers capable of endovascular therapy (47). Furthermore, interhospital transfer pathways for patients requiring thrombectomy are not yet standardized in many regions, contributing to additional delays.

Our finding that only a minority of patients utilized EMS (“calling 120”) highlights a critical gap in the prehospital treatment chain. Strengthening the interface between community recognition, EMS activation, and hospital prenotification represents a priority for reducing overall delay. Future interventions should target not only patient-level factors identified in this study but also system-level improvements, including standardized EMS training, stroke-ready ambulance designations, and regional stroke network development.

The findings suggest that targeted, multi-level interventions—such as health education campaigns, optimization of emergency medical resources, and development of community support systems—can effectively reduce delay times, increase treatment rates (e.g., thrombolysis), and improve patient outcomes.

Our findings on the factors influencing pre-hospital delay should be considered within the broader context of prognostic determinants for cerebral infarction outcomes. A comprehensive understanding of prognosis (48, 49), encompassing both pre-hospital and in-hospital phases, is crucial for developing effective stroke care strategies.

It should be clarified that while we use the term “prehospital delay” in its conventional sense to denote the interval from symptom onset to hospital arrival, our study did not assess EMS system processes such as dispatch, transport, or prehospital triage. Readers should interpret our findings within the context of patient- and caregiver-level determinants.

## Data Availability

The original contributions presented in the study are included in the article/supplementary material, further inquiries can be directed to the corresponding author.
